# Whole-Body Imaging for the Primary Staging of Melanomas—A Single-Center Retrospective Study

**DOI:** 10.3390/cancers15215265

**Published:** 2023-11-02

**Authors:** Kristine E. Mayer, Jochen Gaa, Sophia Wasserer, Tilo Biedermann, Oana-Diana Persa

**Affiliations:** 1 Clinic and Policlinic for Dermatology and Allergology, Technical University Munich, 80802 Munich, Germany; 2 Institute for Diagnostic and Interventional Radiology, Technical University Munich, 81675 Munich, Germany

**Keywords:** PET-CT, melanoma, staging, imaging, diagnosis

## Abstract

**Simple Summary:**

In melanoma, distant metastasis is frequent. To assess the state of metastasis formation at diagnosis, a common method employed is an invasive sentinel lymph node biopsy. However, in recent years, the use of non-invasive positron emission tomography combined with computed tomography (PET/CT) imaging for primary staging, especially in thick primary melanoma, has significantly increased. This study aims to elucidate the value of whole-body imaging for staging at melanoma diagnosis and to identify when whole-body imaging is currently used for primary staging, as well as evaluating its diagnostic precision. Furthermore, its effects on the subsequent diagnostic and therapeutic procedures should be determined to better understand the possible future implications.

**Abstract:**

Background: Melanoma staging at diagnosis predominantly depends on the tumor thickness. Sentinel lymph node biopsy (SLNB) is a common tool for primary staging. However, for tumors of >4 mm with ulceration, 3D whole-body imaging and, in particular, Fluor-18-Deoxyglucose positron emission tomography combined with computed tomography (^18^F-FDG-PET/CT), is recommended beforehand. This study aimed to investigate the real-world data of whole-body imaging for initial melanoma staging and its impact on the subsequent diagnostic and therapeutic procedures. Methods: In this retrospective single-center study, 94 patients receiving ^18^F-FDG-PET/CT and six patients with whole-body computed tomography (CT) scans were included. The clinical characteristics, imaging results, and histologic parameters of the primary tumors and metastases were analyzed. Results: Besides the patients with primary tumors characterized as pT4b (63%), the patients with pT4a tumors and pT3 tumors close to 4 mm in tumor thickness also received initial whole-body imaging. In 42.6% of the patients undergoing ^18^F-FDG-PET/CT, the imaging results led to a change in the diagnostic or therapeutic procedure following on from this. In 29% of cases, sentinel lymph node biopsy was no longer necessary. The sensitivity and specificity of ^18^F-FDG-PET/CT were 66.0% and 93.0%, respectively. Conclusion: Whole-body imaging as a primary diagnostic tool is highly valuable and influences the subsequent diagnostic and therapeutic procedures in a considerable number of patients with a relatively high tumor thickness. It can help avoid the costs and invasiveness of redundant SLNB and simultaneously hasten the staging of patients at the time of diagnosis.

## 1. Introduction

New findings have altered the therapeutic course after melanoma diagnosis dramatically over the last decade. Apart from surgery, non-invasive therapeutic options including immune checkpoint inhibitor treatment, targeted therapy, and radiation for distant disease have been established, benefitting large cohorts of melanoma patients. Meanwhile, the recommendations for staging in malignant melanoma have barely changed. The diagnostic procedure employed is based on the risk of metastatic spread mainly determined by the thickness and ulceration of the primary tumor and categorized by the American Joint Committee on Cancer (AJCC) classification [1,2]. Sentinel lymph node biopsy (SLNB) is very commonly used for melanoma primary staging because of its high sensitivity [3], allowing for the early detection of lymph node metastasis, and because of its important prognostic value in melanoma [4]. After the removal of a positive sentinel lymph node, adjuvant systemic therapy with an immune checkpoint inhibitor or targeted therapy is recommended [5,6,7]. However, for high-risk patients with a primary tumor thickness of >4 mm and ulceration, namely, pT4b tumors, the recommendation is to perform three-dimensional (3D) imaging prior to SLNB [8,9].

Here, Fluor-18-Deoxyglucose positron emission tomography combined with computed tomography (^18^F-FDG-PET/CT) is the tool of choice. Currently, ^18^F-FDG-PET/CT is routinely used in monitoring the follow-up results of melanoma patients, taking advantage of its high negative predictive value [10]. Especially in patients with advanced melanoma undergoing systemic treatment [11], it offers an accurate measurement of the tumor burden in the form of the metabolic tumor volume [12,13]. In comparison to computed tomography (CT) only, ^18^F-FDG-PET/CT combines morphological information with functional data resulting from glucose uptake in highly metabolically active tissue, thus leading to the highest sensitivity possible in comparison to each imaging method on its own [14,15]. Moreover, further technological developments have broadened the diagnostic potential of this non-invasive imaging method. In particular, the combination and correlation of findings from ^18^F-FDG-PET and CT have significantly increased the sensitivity [15]. Furthermore, technological advances such as small-voxel reconstruction and novel reconstruction algorithms have simplified the detection of in-transit metastasis [16,17,18], and have improved the diagnostic scope.

Micrometastasis, however, can still be detected more accurately by SLNB [19,20,21]. For ^18^F-FDG-PET imaging, the proportion of correctly detected lymphatic metastasis drops dramatically from 83–100% to only 23% when the size of the lymphatic metastasis is smaller or equal to 5 mm [22]. Because the different levels of likelihood of obtaining a positive finding depend on the tumor thickness [23] and the limited sensitivity in initial nodal metastasis detection [24], whole-body 3D imaging for initial staging is only recommended for patients with a higher tumor thickness, namely primary-stage pT4b tumors [6]. Still, it has not yet been comprehensively applied for primary staging in melanoma patients. A survey from Germany in 2018 and 2019 revealed that only 16.8% of eligible patients received prior ^18^F-FDG-PET/CT imaging [25].

The aim of this retrospective study, therefore, was to better understand the diagnostic value of primary whole-body PET/CT imaging. In this study, a special focus was placed on the changes in the clinical course of action based on the PET/CT results obtained.

## 2. Methods

The study was approved by the ethics committee of the Faculty of Medicine of the Technical University of Munich (TUM). All melanoma patients treated at the Klinikum rechts der Isar, TUM, between February 2015 and January 2023 were screened using the staging method immediately after diagnosis. In total, 100 patients received whole-body imaging as the primary staging tool and were included in this single-center retrospective study. Clinical data including age, gender, type of melanoma, tumor thickness, the histologic characteristics of the primary tumor, and imaging results were collected from all patients. In total, 94 patients underwent ^18^F-FDG-PET/CT. A further six patients received whole-body CT imaging only (Table 1), even though PET/CT had been favored. This was mainly due to organizational issues or the patient’s will. The ^18^F-FDG-PET/CT and CT scans were examined by at least two independent medical doctors trained in radiology and nuclear medicine. Based on the results, the subsequent diagnostic and therapeutic procedures were determined by an interdisciplinary board for skin cancer.

First, the characteristics of the cohort receiving primary whole-body imaging were analyzed descriptively. In particular, the aim was to evaluate clinical features leading to the decision for whole-body imaging in melanoma patients after diagnosis. These included tumor thickness, ulceration of the primary tumor, clinical suspicion of distant metastasis, and the specific type of acrolentiginous melanoma. Next, the final staging results according to the AJCC classification were determined. In particular, it was necessary to characterize the type of metastases detected, such as locoregional or distant lymphatic metastasis and other types of distant metastases. Moreover, the impact of whole-body imaging on the subsequent diagnostic and therapeutic procedures was analyzed. Reasons for an alteration in the procedure employed were identified and grouped into the following categories: altered surgical procedure, neoadjuvant therapy, and declined surgical procedure and subsequent diagnostics because of the suspicion of a secondary malignancy. Moreover, it was of special interest to analyze if SLNB was still performed after imaging. The patient characteristics matched with the respective imaging results of all patients are presented in Supplementary Tables S1 and S2. Supplementary Table S1 summarizes patients with melanoma-associated findings and Supplementary Table S2 summarizes patients without any melanoma-associated findings.

To evaluate sensitivity and specificity as well as positive and negative predictive values in this cohort for primary ^18^F-FDG-PET/CT imaging, PET/CT scans and the conclusions drawn thereof were compared with clinical follow-up data. The latter included additional imaging findings from intermediate stagings, histological findings from SLNB, and lymph node dissections and metastasis surgery as well as further clinical documentation on the patients. Finally, the size of the lymphatic metastases missed by ^18^F-FDG-PET/CT was analyzed descriptively to gain an impression of the limit of detection of the imaging method in this setting.

The data obtained were analyzed and visualized with Microsoft Office, BioRender, and GraphPad Prism 9 software.

## 3. Results

### 3.1. Indication for Whole-Body Imaging

**Figure 1:**
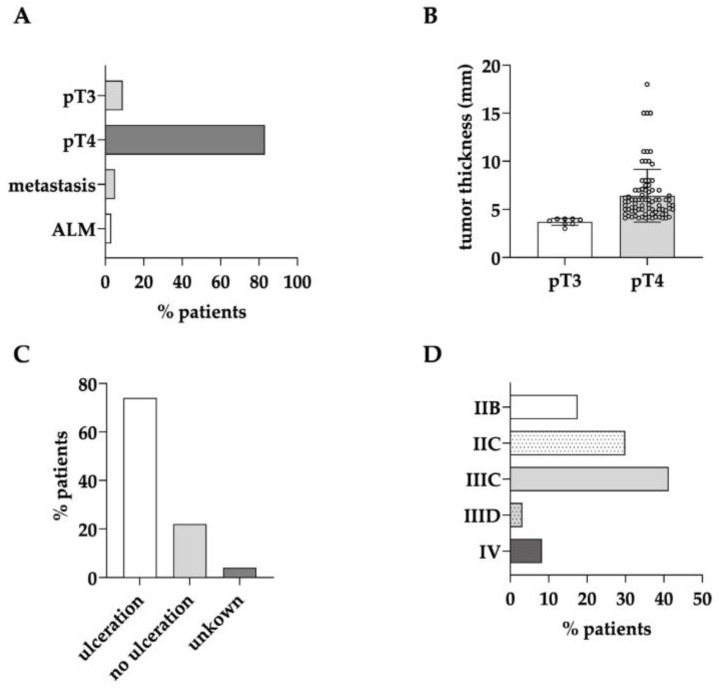
Indication for whole-body imaging as the primary staging procedure in melanoma patients. High tumor thickness of the primary tumor and clinical or sonographic suspicion of metastasis or acrolentiginous melanoma of unknown or undeterminable tumor thickness (ALM) were indications for primary whole-body imaging in melanoma patients (**A**). Tumor thickness of primary pT3 and pT4 tumors (mean ± sd) in (**B**) and ulceration of the primary tumor (**C**) are relevant indicators for primary whole-body staging. The final staging results of all patients under investigation are shown in (**D**).

In total, 100 patients who underwent whole-body imaging for primary melanoma staging were included in this retrospective study. The patients’ characteristics are summarized in Table 1 and Supplementary Tables S1 and S2. In general, primary whole-body imaging with ^18^F-FDG-PET/CT or whole-body CT in the absence of clinically suspected metastasis is mainly recommended for melanoma patients with pT4b primary tumors [8]. Correspondingly, the majority of patients from the identified cohort had a primary tumor of >4 mm tumor thickness and with ulceration (Table 1, Supplementary Tables S1 and S2). By including 63% of all patients, this group was also the largest (Figure 1A). However, whole-body imaging was also performed on a substantial amount of the patients with pT4a tumors without ulceration (19%) and even pT3 tumors close to the limit of 4 mm tumor thickness (9%; Figure 1A,B). Single patients received primary whole-body imaging because lymphatic metastasis was suspected after sonography or because they had acrolentiginous melanoma of unknown or undeterminable tumor thickness (Figure 1A). In total, ulceration as a dominant risk factor was present in almost three fourths of the melanoma patients receiving primary whole-body imaging (Figure 1C). After staging was completed, in 44% of the patients, locoregional metastasis was found, and in 9% of the patients, distant metastasis was found (Figure 1D).

### 3.2. Results of Whole-Body ^18^F-FDG-PET/CT Imaging

**Figure 2:**
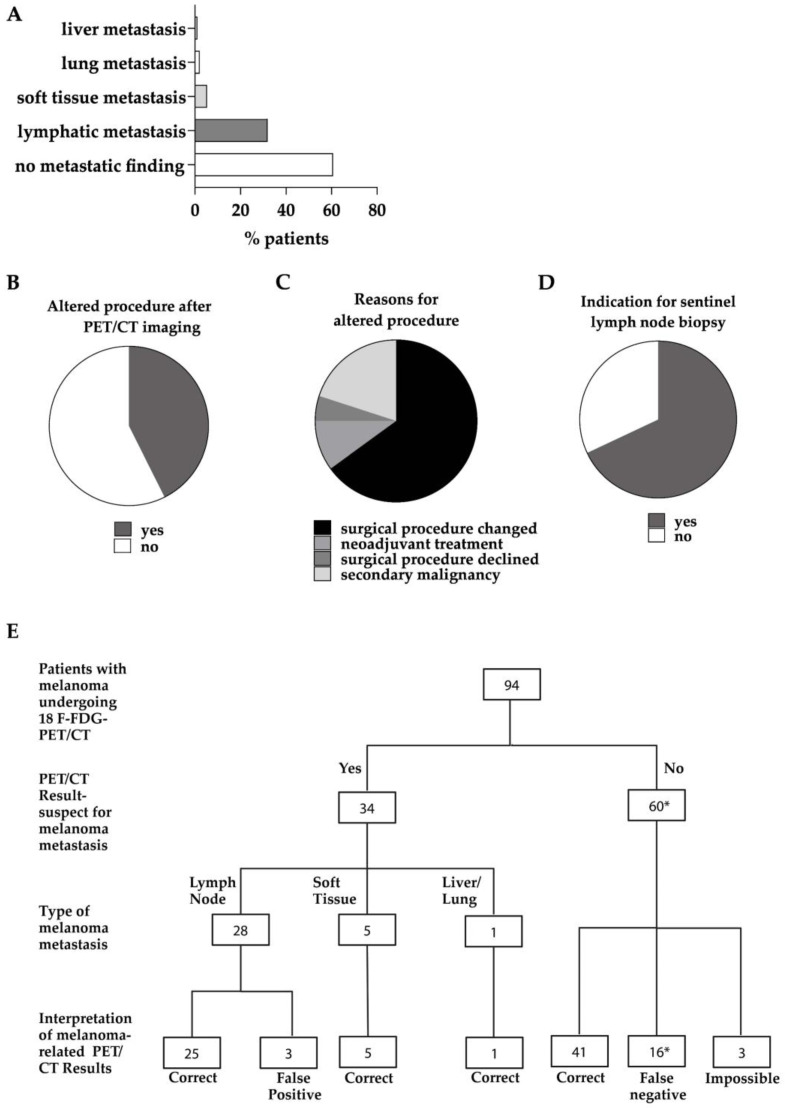
Results of ^18^F-FDG-PET/CT imaging and implications for the subsequent diagnostic procedure. (**A**) shows the ^18^F-FDG-PET/CT results categorized as no metastatic finding or different metastatic manifestations. The proportion of altered procedures after ^18^F-FDG-PET/CT imaging (**B**) is divided into altered surgical procedure, neoadjuvant therapy, and declined surgical procedure and further diagnostics because of the suspicion of a secondary malignancy (**C**). (**D**) depicts the rate of indication for sentinel lymph node biopsy. (**E**) summarizes the ^18^F-FDG-PET/CT results and their final interpretation. One patient had an unclear pulmonary nodule, compatible with metastasis, but histologically identified as carcinoid and no indication of lymph node metastasis, but micrometastasis in SLNB (indicated by *). The patient was excluded for the calculation of the positive and negative predictive value.

**Figure 3:**
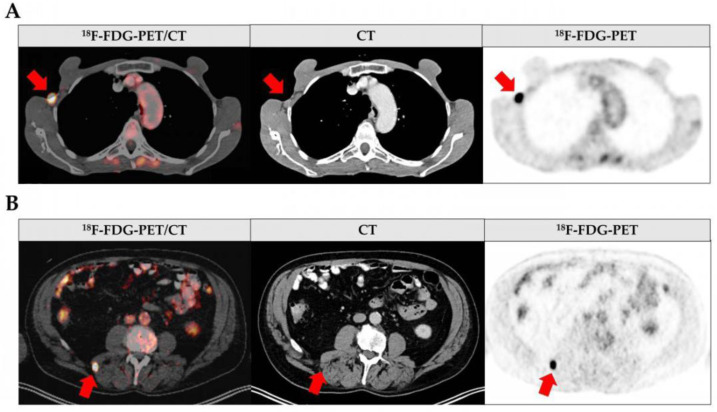
Images from ^18^F-FDG-PET/CT at melanoma diagnosis. Fused ^18^F-FDG-PET/CT (**left**), CT (**middle**) and ^18^F-FDG-PET (**right**) scans. Metastases are indicated by red arrows. (**A**) shows an axillary lymphatic metastasis in a 55-year-old patient and (**B**) depicts a metastasis in the autochthonous back muscles in a 66-year-old patient.

Overall, 63.8% of all whole-body ^18^F-FDG-PET/CT scans showed no sign of metastatic disease (Figure 2A). The most frequent site for metastasis was the lymph nodes, followed by soft tissue metastasis, lung metastasis, and liver metastasis in just one case (Figure 2A, Supplementary Table S1). The exemplary ^18^F-FDG-PET/CT scans showing lymph node and soft tissue metastasis are depicted in Figure 3. Based on the ^18^F-FDG-PET/CT results, the subsequent diagnostic or therapeutic procedure was altered in 40 out of the 94 cases (42.6%; Figure 2B). In most cases, the type of subsequent surgical intervention changed (65%; Figure 2C). Namely, locoregional lymph node dissection, the targeted resection of suspect lymph nodes, and the resection or biopsy of soft tissue or other distant metastases were performed. Four patients instead received neoadjuvant immunotherapy (10%), while two patients refused surgery after imaging (5%; Figure 2C). In 20% of the cases with an altered procedure, this was due to the first diagnosis of a secondary malignancy either directly suspected upon interpretation of the ^18^F-FDG-PET/CT scans or histologically verified after initially being considered melanoma metastasis (Figure 2C). The histology revealed two cases of non-Hodgkin lymphoma, two cases of prostate carcinoma, one case of colon carcinoma as part of Lynch syndrome, one case of mamma carcinoma, one case of carcinoid tumor, and one case of giant cell tumor.

Overall, the performance of ^18^F-FDG-PET/CT imaging made SLNB obsolete in almost one third of all patients (29%; Figure 2D, Supplementary Table S1). When SLNB was performed, it revealed micrometastasis in 42.4% of patients. Moreover, ^18^F-FDG-PET/CT imaging for primary staging changed the consequent therapeutic decision in 28.7% of all the patients undergoing imaging.

Figure 2E categorizes the imaging results from the 94 patients undergoing ^18^F-FDG-PET/CT according to their clinical and histological follow-up results.

### 3.3. Comparison of ^18^F-FDG-PET/CT Imaging with Histologic Findings and Follow-Up

The interpretation of the ^18^F-FDG-PET/CT scans based on the metabolic and morphological parameters correctly identified metastasis in 31 cases (Figure 2E). Actual metastasis was present in 47 cases, which was confirmed by histology or clinical course. This resulted in a sensitivity of 66.0% (Table 2). However, in 7 of the 31 cases, not all single lymphatic metastases were identified precisely. The specificity was determined to be 93.2% (Table 2). Similarly, the percentage of patients with melanoma-associated metastasis was high when ^18^F-FDG-PET/CT indicated metastasis (31 out of 34 patients; positive melanoma-associated predictive value: 91.2%; Supplementary Table S1). In two of the falsely presumed cases, ^18^F-FDG-PET suspected lymphatic metastasis because of increased metabolic activity, but a respective CT correlate was missing. In one other case, lymphatic metastasis was assumed because of the round morphology of a lymph node with moderately increased metabolic activity. The negative predictive value of ^18^F-FDG-PET/CT imaging was determined to be 73.2% (Table 2; Supplementary Table S2).

**Figure 4:**
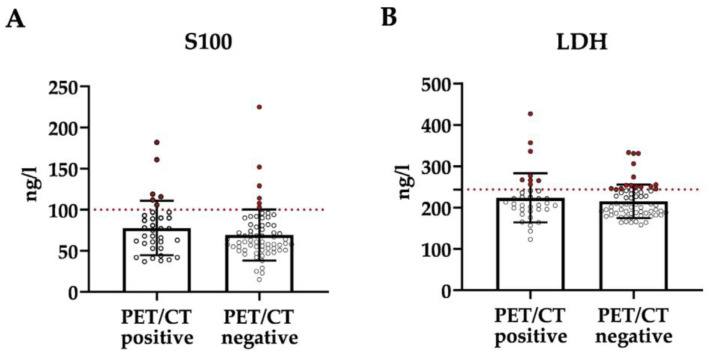
S100 and LDH serum concentrations and PET/CT results. S100 (**A**) and LDH (**B**) serum levels in ng/L are depicted for patients with melanoma-associated positive and negative PET/CT results (*p* > 0.05). Values above the reference levels (S100: 100 ng/L; LDH: 244 ng/L) are shown in red.

Furthermore, the likelihood of elevated serum tumor markers against the PET/CT results was analyzed (Figure 4). Apart from a slight trend towards higher serum concentrations of S100, which was statistically not significant, there was no indication for a correlation between the tumor marker concentration and the PET/CT results.

Finally, the size of the lymphatic metastases missed by ^18^F-FDG-PET/CT was further analyzed. It ranged from single cells detected only by immunohistochemistry (IHC) to a maximum size of 8.3 mm. In the latter case, the metabolic activity shown on the ^18^F-FDG-PET/CT images was slightly increased without a respective CT correlate, which was interpreted as non-metastatic because of missing strong criteria indicating metastasis. In another patient who also suffered from non-Hodgkin lymphoma, which was suspected upon examination of imaging because of a generalized lymph node enlargement, the identification of a micrometastasis resulting from the malignant melanoma was hampered, leading to no correct identification of a micrometastasis of 2.3 mm in size. The rest of the missed micrometastases were smaller than or equal to 2.0 mm.

## 4. Discussion

By combining metabolic and morphological information, ^18^F-FDG-PET/CT is a powerful tool for detecting metastasis in oncology and melanoma staging (Figure 3) [14]. The value of this imaging method is widely accepted for the detection of distant metastases [20]. Lymph node metastases, however, are assumed to be reliably detectable only at a size of 6 mm and larger [22]. Since lymph node metastases are the most frequent and earliest form of metastasis in malignant melanoma, their detection is crucial in melanoma staging. This raises the question as to whether whole-body imaging is nevertheless suitable for initial melanoma staging as it is recommended for patients from AJCC stage IIC (>4 mm tumor thickness and ulceration) onwards [9].

Screening the TUM melanoma patients from the last eight years revealed that 100 patients underwent whole-body imaging for primary staging. This retrospective cohort consisted mainly of AJCC stage IIC patients. However, it was observed that patients with >4 mm tumor thickness but no ulceration (pT4a) and smaller tumor thickness also received whole-body imaging (Figure 1A). The pT3 tumors were also of a rather high tumor thickness, close to or at 4 mm (Figure 1B). Overall, the majority of the primary tumors from patients receiving whole-body staging were ulcerated (Figure 1C). In three patients, staging with PET/CT was indicated individually in patients with acrolentiginous melanoma where the tumor thickness was undeterminable or smaller than 3 mm (Figure 1A, Supplementary Table S2). In one of them, popliteal and inguinal lymph node metastases were detected on the PET/CT scan. The respective high sensitivity and specificity values for the whole group (Table 2) may therefore indicate that individual decisions for initial ^18^F-FDG-PET/CT staging in selected patients with a higher risk such as thick pT3 tumors, pT4a tumors, and acrolentiginous melanoma are reasonable.

Overall, distant metastasis (stage IV) was identified in 9% of the patients (Figure 1D), with soft tissue metastasis being the most frequent form of distant metastasis and liver and lung metastasis being detected only in single cases (Figure 2A). Because of the small number of cases with respective forms of metastasis, an evaluation of the benefit of ^18^F-FDG-PET/CT in this context is highly restricted. Therefore, larger studies with several hundreds of patients would be needed.

Nevertheless, only 63.8% of the ^18^F-FDG-PET/CT scans from the cohort analyzed were without any pathological findings (Figure 2A). Correspondingly, the ^18^F-FDG-PET/CT results led to an alteration in the subsequent procedure employed in a considerable number of patients (42.6%) (Figure 2B). Based on the imaging results, the surgical procedure in particular was altered, and alternative therapeutic strategies such as adapted surgical techniques and neoadjuvant immunotherapy were able to be exploited (Figure 2C). In this cohort, the procedure was altered due to the detection of a secondary malignancy in about 1/5 of the patients (Figure 2C), which seems a rather large proportion and is not described in similar studies. After whole-body imaging, SLNB was obsolete in 29% of patients (Figure 2D). From the 76 pT4b patients receiving ^18^F-FDG-PET/CT imaging described by Hardie and colleagues, only 21% demonstrated potential or actual metastasis, with only 18% of the patients experiencing altered clinical care [26]. Since Hardie and colleagues do not provide any diagnostic validity parameters, such as sensitivity and specificity, which are expected to be lower than in this study, a comparison remains unavailable.

The sensitivity and specificity of ^18^F-FDG-PET/CT imaging in this retrospective study of preselected patients were determined to be 66.0% and 93.0%, respectively. This is in line with a meta-analysis from Schröer-Günther and colleagues that describes similar ranges for the sensitivity and specificity of ^18^F-FDG-PET/CT in patients of AJCC stage III and IV [27].

It has been reported that the sensitivity and specificity of ^18^F-FDG-PET/CT also depend on the examined organ [28]. Due to the small number of patients with distant metastases in this study, separate validity parameters could not be calculated. The most frequent form of distant metastasis were soft tissue metastases, which were detected reliably. As mentioned above, a larger study population is needed to determine the validity parameters, also on an organ-based level in this context of primary staging. Because the detection of very early lymphatic metastasis, which can sometimes only be detected by immunohistochemistry, is insufficient, the sensitivity of ^18^F-FDG-PET/CT in thinner melanoma is limited [11,19,22,24]. Accordingly, the overall high sensitivity in this study is favored by the preselection of patients with higher risk by the physicians indicating initial whole-body imaging. On the other hand, the study group consisted not only of patients meeting the recommendations but also others with an individual indication, as described above. The beneficial melanoma-associated validity parameters (Table 2) may therefore justify the physician’s decision based on the individual patient’s risk profile. For this, classical histological parameters like tumor thickness and ulceration [29] were identified as being decisive here. Serum tumor markers, however, could not predict the imaging results (Figure 4). In a prospective pilot study from Stahlie and colleagues on lymph node ultrasound testing and ^18^F-FDG-PET/CT prior to SLNB, patients with AJCC stage IIB and IIC tumors were included [21]. In this small study with only 23 patients, the sensitivity of ^18^F-FDG-PET/CT was very low at only 29%, while the specificity and positive predictive value were determined to be 100% [21]. The large difference in this study may be explained by the higher reliability of the larger cohort and the individual preselection by the physician based on risk leading to the inclusion of pT3b tumors with tumor thicknesses at the upper end of the stated range (Figure 1B). In the future, patient stratification based on risk may be more detailed when additional prognostic biomarkers are identified and transferred to the clinic [30]. This could finally help to decide on which patient should receive whole-body imaging for initial staging. However, to verify the benefit of ^18^F-FDG-PET/CT for patients based on individual physician decisions or the use of biomarkers, larger studies including prospective trials as well are required in the future.

Contrary to the expectation that metastases smaller or equal to 5 mm are highly difficult to detect [22], the size of lymphatic metastases missed by ^18^F-FDG-PET/CT was astonishingly small, most likely due to technical advances over the last two decades. Apart from two cases of 8.3 and 2.3 mm micrometastases which had a PET correlate only or were difficult to assess because of co-occurrence with non-Hodgkin lymphoma, all other missed lymphatic metastases were smaller or equal to 2.0 mm. To further increase the sensitivity, Bärwolf and colleagues proposed to carry out breath-hold imaging to improve the quantitative evaluations [31]. This technique may also avoid further ambiguous interpretations of opposing information from PET and CT scans.

Nevertheless, SLNB remains the more sensitive method for detecting lymphatic micrometastasis. However, the therapeutic landscape of melanoma is changing, with systemic therapies increasingly recommended in earlier stages of disease or in neoadjuvant settings. In this context, non-invasive and repeatable imaging techniques such as ^18^F-FDG-PET/CT may be a suitable diagnostic counterpart. For example, pembrolizumab is now approved as an adjuvant therapy in stage IIB and IIC melanomas, thus putting the performance of SLNB into question. Instead, only the performance of ^18^F-FDG-PET/CT imaging enabling the diagnosis of distant metastasis may influence the therapeutic procedure, making it the more reasonable tool for staging. Overall, with the evolving therapeutic options that are available, the diagnostic toolbox will change. As shown here, ^18^F-FDG-PET/CT imaging already plays a key role in diagnosis nowadays, exceeding the classical indication in primary staging, and as such, this influence is expected to increase in the process of further therapeutic developments.

## 5. Conclusions

This retrospective study highlights the fact that PET/CT imaging may be a sensitive tool for the detection of lymphatic and distant metastasis in melanoma at the time of diagnosis. In a substantial number of patients, the PET/CT findings led to alternative diagnostic and therapeutic procedures being employed. Specifically, it made sentinel lymph node biopsy obsolete in almost one third of the patients undergoing PET/CT imaging. Non-invasive PET/CT may therefore be an attractive tool to hasten the initial staging and avoid unnecessary additional SLNB. However, this needs to be confirmed by additional larger and prospective studies.

## Figures and Tables

**Table 1:** Patient characteristics.

Characteristic	Cohort (*n* = 100)
Imaging method	
18F-FDG-PET/CT	94
CT	6
Age	
Median ± SD	65.2 ± 15.9
Range	4–87
Gender	
Female	47
Male	53
Type of melanoma	
Skin	97
Mucosal	3
Tumor thickness	
Mean ± SD (mm)	6.03 ± 2.75
≥4 mm	85
<4 mm	10
not definable	2
Histology of primary	
Ulceration	74
Micrometastasis	11
Lymphatic infiltration (L1)	14
Vascular infiltration (V1)	
Tumor staging (AJCC)	
Stage II	47
IIB	17
IIC	29
Stage III	44
IIIC	40
IIID	3
Stage IV	9

**Table 2:** Parameters of the diagnostic melanoma-associated validity of ^18^F-FDG-PET/CT.

Parameter		Correctly Identified	Cohort Size
Sensitivity	66.0%	31	47
Specificity	93.2%	41	44
Positive predictive value	91.2%	31	34
Negative predictive value	73.2%	41	56

## Data Availability

Data available within the article or its Supplementary Materials.

## Supplementary Materials

The following supporting information can be downloaded at: https://www.mdpi.com/article/10.3390/cancers15215265/s1, Table S1: Patients with melanoma-associated positive findings in primary staging; Table S2: Patients without any melanoma-associated findings in primary staging.
