# Supplementary material for: Whole-Body Imaging for the Primary Staging of Melanomas—A Single-Center Retrospective Study
Source: Cancers (Basel). 2023 Nov 2;15(21):5265. doi: 10.3390/cancers15215265 (PMC10648596; doi:10.3390/cancers15215265)
Supplement: Supplementary file 1 [file cancers-15-05265-s001.zip › Suppl Table 1 PET.pdf]

Supplementary Table 1. Patients with melanoma-associated positive findings in primary staging

Nr.	Sex	Age	Tumor Thickness	Ulceration	Indication PET	AJCC	Localisation Primary	Tumor Marker*	PET: Metastasis LN	ST	Other	Localisation Of Metastasis	PET Positive	CT Positive	SLNB Conducted	SLNB Result	PET Result Compared To Histological Or Clinical Follow-up	LAD	Other Surgery	ICI	Targeted Therapy	IFN	Radiation
1	f	55	>15	yes	pT4b	IIIB	trunk	elevated	x			axillary	x	x	no		correct	x		x			
2	f	35	5.4	yes	pT4b	IIIC	trunk	normal	x			axillary	x	x	no		correct	x				x	x
3	f	51	5.5	yes	pT4b	IIIC	lower leg	normal	x			inguinal	x	x	yes	positive	correct			x			
4	m	43	7.0	yes	pT4b	IIIC	trunk	normal	x			axillary	x	x	no		correct	x		x			
5	m	45	2.8	no	###	IIIC	trunk	normal	x			axillary	x	x	yes	positive	partially correct			x			
6	m	80	2.2	yes	###	IIIC	trunk	normal	x			axillary	x	x	no		correct	x			x		x
7	f	48	>6	yes	pT4b	IIID	trunk	normal	x			axillary	x	(x)	yes	positive	correct				x		
8	m	73	7.5	yes	pT4b	IIIC	trunk	normal	x			axillary	(x)	x	yes	positive	partially correct				x		x
9	f	75	11	yes	pT4b	IIID	foot	elevated	x			inguinal	x	x	no		correct	x		x			x
10	f	72	3.1	yes	###	IIIC	foot	elevated	x			inguinal	x	x	no		correct	x		x			
11	m	80	3.6	yes	###	IIIC	knee	normal	x			inguinal	x	x	no		correct		x	x			
12	m	39	>8	yes	pT4b	IIIC	trunk	normal	x			inguinal	x	x	no		partially correct		x	x			
13	m	77	2.5	no	ALM	IIIC	foot	normal	x			popliteal + inguina	x	x	no		partially correct	x		x			
14	f	71	>10	no	pT4a	IIIC	upper arm	elevated	x			axillary	x	x	no		correct	x		x			x
15	m	70	4.5	yes	pT4b	IIIC	trunk	elevated	x			axillary	x	x	no		partially correct		x	x			
16	f	55	>7	yes	pT4b	IV	lower leg	normal	x			inguinal + iliacal	x	x	no		correct		x	x			
17	f	75	4.5	yes	pT4b	IIIB	finger	elevated	x			axillary	x	x	no		correct	x		x			
18	f	82	##	##	###	IVA	mucosal	elevated	x			jaw angle	x	x	no		correct	x		x			
19	m	73	7.5	yes	pT4b	IIIC	trunk	normal	x			axillary	x	x	yes	positive	partially correct			x			
20	f	82	18	yes	pT4b	IIID	trunk	elevated	x			axillary	x	x	no		correct	x		x			x
21	f	54	5.2	no	pT4a	IIIC	scalp	normal	x			cervical	x	x	no		correct	x	x	x			
22	m	69	>11	yes	pT4b	IV	trunk	normal	x			inguinal	x	x	no		correct		x				
23	m	78	6.2	yes	pT4b	IIIC	trunk	normal	x			axillary	x	x	yes	positive	correct			x			
24	m	52	7.0	yes	pT4b	IIIB	upper leg	normal	x			inguinal	x	x	no		correct	x		x			x
25	m	75	6.5	no	pT4a	IIIC	lower leg	normal	x			popliteal + inguina	x	x	yes	positive	correct			x			
26	f	68	6.0	yes	pT4b	IIC	trunk	normal	x			axillary	(x)	x	no		false positive	x					
27	m	56	>10	yes	pT4b	IIC	trunk	normal	x			axillary	x	0	yes	negative	false positive			x			
28	f	74	4.7	no	pT4a	IIB	trunk	normal	x			axillary	x	0	no		false positive						
29	m	77	5.6	yes	pT4b	IV	trunk	normal			x	lung, liver	x	x	no		correct			x			
30	m	77	5.0	yes	pT4b	IV	face	normal		x		parotis	x	x	no		correct	x		x			x
31	m	44	>6	yes	pT4b	IV	trunk	elevated		x		thorax	x	x	no		correct		(x)		x		x
32	m	66	#	#	pT3	IV	mucosal	normal		x		muscle of back skin, fat of	x	x	no		correct		x	x			
33	f	78	7.0	yes	pT4b	IIIC	scalp	normal		x		retroauricular	x	x	no		correct		x	x			x
34	m	78	>5	yes	pT4b	IIIC	face	elevated		x		cheek	x	x	no		correct		x	x			
(35)	m	57	5	yes	pT4b	IIIC	upper leg	normal	x			inguinal		x	yes		partially correct			x			

* LDH or S-100 above reference values, LN lymph node metastasis, ST soft tissue metastasis, LAD lymphadenectomy, ICI immune-checkpoint inhibitior therapy, IFN Interferon, # mucosal melanoma, ## unknown, ### clinical suspicion of lymph node metastasis, ALM acrolentiginous melanoma, (x) planned, ( ) CT only
