# Supplementary material for: Whole-Body Imaging for the Primary Staging of Melanomas—A Single-Center Retrospective Study
Source: Cancers (Basel). 2023 Nov 2;15(21):5265. doi: 10.3390/cancers15215265 (PMC10648596; doi:10.3390/cancers15215265)
Supplement: Supplementary file 1 [file cancers-15-05265-s001.zip › Suppl Table 2 PET.pdf]

Supplementary Table 2. Patients without any melanoma-associated findings in primary staging

Nr.	Sex	Age	Tumor Thickness	Ulceration	Indication PET	AJCC	Localisation Primary	Tumor Marker*	PET Result	SLNB Result	PET Result Compared To Histological Or Clinical Follow-up
1	m	62	4.2	yes	pT4b	IIC	trunk	normal	negative	negative	correct
2	f	57	3.9	no	pT3a	IIB	upper leg	normal	negative	negative	correct
3	f	70	5.4	yes	pT4b	IIC	lower leg	normal	negative	negative	correct
4	m	78	6.0	yes	pT4b	IIC	lower arm	normal	urinary stasis and prostate cancer	negative	correct
5	m	73	5.8	yes	pT4b	IIC	trunk	normal	negative	negative	correct
6	f	79	4.2	yes	pT4b	IIC	face	normal	negative	negative	correct
7	m	53	4.0	yes	pT3b	IIB	lower leg	normal	negative	negative	correct
8	m	76	>5	##	pT4	IIIC	foot	normal	negative	negative	correct
9	f	58	6.3	yes	pT4b	IV	trunk	normal	negative	-	correct
10	m	51	3.5	yes	pT3b	IIB	trunk	elevated	negative	negative	correct
11	f	49	3.8	yes	pT3b	IIB	trunk	normal	negative	negative	correct
12	m	51	4.3	no	pT4a	IIB	upper arm	elevated	negative	negative	correct
13	m	77	4.1	yes	pT4b	IIC	upper arm	normal	negative	-	correct
14	m	69	10	yes	pT4b	IIC	upper leg	elevated	negative	negative	correct
15	m	55	8	yes	pT4b	IIC	trunk	normal	negative	negative	correct
16	f	74	8	yes	pT4b	IIC	face	elevated	negative	negative	correct
17	f	84	4.2	yes	pT4b	IIC	face	elevated	negative	negative	correct
18	m	77	4.1	no	pT4a	IIB	trunk	normal	negative	negative	correct
19	m	80	15	no	pT4a	IV	trunk	normal	negative	-	correct
20	m	33	6.8	no	pT4a	IIB	trunk	normal	negative	negative	correct
21	m	78	5.1	yes	pT4b	IIc	face	normal	negative	negative	correct
22	f	68	#	yes	pT4b	IIC	mucosal	normal	negative	negative	correct
23	m	80	5.4	no	pT4a	IIB	scalp	elevated	negative	-	correct
24	f	82	5	yes	pT4b	IIC	upper arm	normal	negative	negative	correct
25	m	79	9.7	yes	pT4b	IIC	trunk	normal	negative	-	correct
26	f	52	5.5	no	pT4a	IIB	foot	normal	negative	negative	correct
27	f	63	>6	yes	pT4b	IIC	lower leg	normal	negative	negative	correct
28	m	85	>7	yes	pT4b	IIC	neck	normal	negative	-	correct
29	m	79	>5	yes	pT4b	IIIC	trunk	normal	negative	negative	correct
30	f	71	4.2	no	pT4a	IIB	hand	normal	negative	negative	correct
31	m	62	##	yes	ALM	II	foot	elevated	negative	negative	correct
32	f	73	4.3	yes	pT4b	IIC	foot	normal	negative	negative	correct
33	f	73	3.5	yes	pT3b	IIB	foot	elevated	negative	negative	correct
34	m	48	5.1	yes	pT4b	IIC	upper leg	normal	negative	negative	correct
35	f	78	4.8	no	pT4a	IIB	scalp	normal	negative	-	correct
36	f	4	4.1	no	pT4a	IIB	lower leg	elevated	negative	-	correct
37	f	51	4.9	yes	pT4b	IIC	upper leg	elevated	negative	negative	correct
38	m	80	5.4	no	pT4a	IIC	scalp	elevated	negative	-	correct
39	f	84	4.1	yes	pT4b	IIC	lower leg	normal	mamma carcinoma	-	correct
40	f	82	6	yes	pT4b	IIC	upper arm	normal	multiple axillary, retro- and interpectoral lymph nodes as metastases from mamma carcinoma	-	correct
41	m	23	6.0	yes	pT4b	IIC	lower leg	normal	colon carcinoma with lynch syndrome	-	correct
42	f	87	4.0	yes	pT3b	IIIC	finger	normal	negative	positive	false negative
43	f	58	5.2	yes	pT4b	IIIC	trunk	normal	negative	positive	false negative
44	f	20	4.8	no	pT4a	IIIC	trunk	normal	negative	positive	false negative
45	m	53	7.1	yes	pT4b	IIIC	trunk	normal	negative	positive	false negative
46	f	56	>3	yes	pT3b, R1	IIIC	lower leg	normal	negative	positive	false negative
47	f	80	>4	yes	pT4b	IIIC	finger	normal	negative	positive	false negative
48	m	68	4.5	yes	pT4b	IIIC	trunk	normal	negative	positive	false negative
49	f	80	##	##	ALM	III	foot	elevated	negative	positive	false negative
50	f	62	6.4	no	pT4a	IIIA	trunk	normal	negative	positive	false negative
51	m	54	15	yes	pT4b	IIIC	upper leg	elevated	negative	positive	false negative
52	f	73	>5	yes	pT4b	IIIC	foot	normal	negative	positive	false negative
53	m	41	5.0	yes	pT4b	IIIC	upper leg	elevated	metabolically active inguinal lymph node without CT correlate	positive	false negative
54	m	80	6.0	yes	pT4b	IIIC	trunk	elevated	generalized multiple suspect lymph nodes and bone marrow expansion, non-Hodgkin lymphoma suspected	positive	false negative
55	m	63	4.5	yes	pT4b	IIIC	lower leg	normal	prostate cancer with single prominent retroperitoneal lymph nodes	positive	false negative
56	f	59	11	yes	pT4b	IIIC	trunk	normal	metabolically active soft tissue tumor on foot	positive	false negative
57	f	58	5.8	no	pT4a	IIIC	foot	elevated	unclear pulmonary nodule, compatible with metastasis, histologically identified as carcinoid	positive	false negative for lymph node metastasis
58	f	79	8	yes	pT4b	IIC	trunk	normal	negative	negative	impossible
59	m	60	5.8	no	pT4a	IIB	trunk	normal	negative	-	impossible
60	f	71	4.5	no	pT4a	IIB	lower leg	normal	negative	-	impossible
(61)	f	57	6	yes	pT4b	IIC	foot	normal	negative	negative	correct
(62)	m	74	4.2	yes	pT4b	IIC	trunk	normal	negative	negative	correct
(63)	m	82	4.0	yes	pT3b	IIB	foot	normal	negative	negative	correct
(64)	m	38	6	yes	pT4b	IIC	trunk	normal	negative	negative	impossible
(65)	m	86	4.1	yes	pT4b	IIIC	trunk	elevated	negative	negative	impossible

* LDH or S-100 above reference values, # mucosal melanoma, ## unknown, ALM acrolentiginous melanoma, ( ) CT only
